#  Design, Synthesis and Anticonvulsant Activity of 2-(2-Phenoxy) phenyl- 1,3,4-oxadiazole Derivatives 

**Published:** 2013

**Authors:** Sayyed Abbas Tabatabai, Saoka Barghi Lashkari, Mohammad Reza Zarrindast, Mohammadreza Gholibeikian, Abbas Shafiee

**Affiliations:** a *Department of Medicinal Chemisty School of Pharmacy, Shahid Beheshti University of Medical Sciences, Tehran, Iran.*; b *Phytochemistry Research Center, Shahid Beheshti University of Medical Sciences, Tehran, Iran.*; c *Department of Pharmacology, Faculty of Medicine, Tehran University of Medical Sciences, Tehran, Iran.*; d *Department of Chemistry, Faculty of Basic Science, Semnan University, Semnan, Iran.*; e *Department of Medicinal Chemistry, Faculty of Pharmacy, Tehran University of Medical Sciences, Tehran, Iran.*

**Keywords:** 1,3,4-Oxadiazole derivatives, Synthesis, Conformational analysis, Anticonvulsant activity

## Abstract

Benzodiazepines are useful drugs for treatment of sleep disorders, anxiety, seizure cases and skeletal muscle cramps. Some derivatives of 2-(2-Phenoxy) phenyl-1, 3, 4-oxadiazole were synthesized as benzodiazepine receptor agonists. Conformational analysis and superimposition of energy minima conformers of the compounds on estazolam, a known benzodiazepine agonist, reveal that the main proposed benzodiazepine pharmacophores were well matched. Anticonvulsant activity of the synthesized compounds, determined by pentylenetetrazole-induced lethal convulsion test, showed that the introduction of an amino substituent in position 5 of 1,3,4- oxadiazole ring generates compound 9 which has a respectable effect. The results are in agreement with SAR of benzodiazepine receptor ligands since the elimination of electronegative substituent in position 2 of phenoxy ring or position 4 of phenyl ring reduces the anticonvulsant activity.

## Introduction

**Figure 1:**
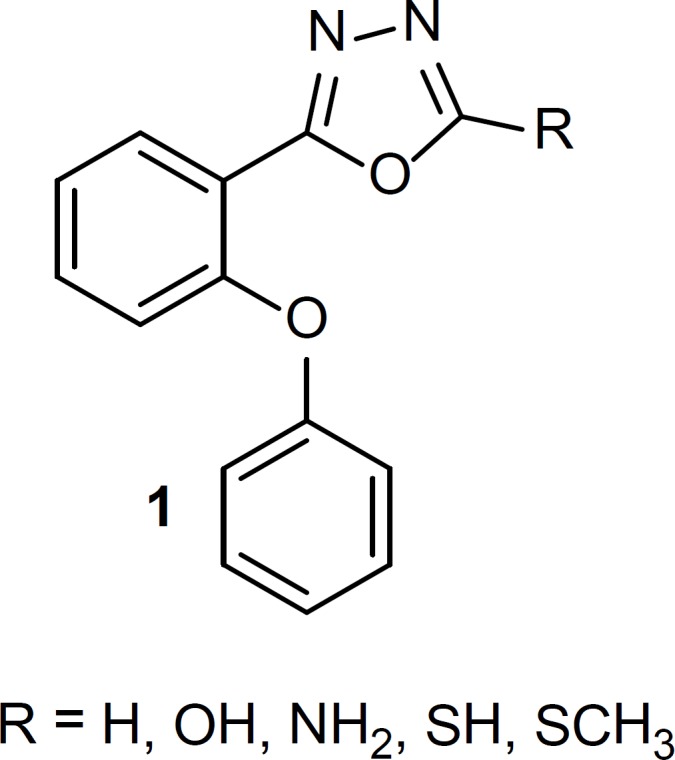
Structure of 1,3,4-oxadiazole derivatives, designed as benzodiazepine receptor ligands

Drugs that interact with benzodiazepine receptors have useful effects on treatment of sleep disorders, anxiety, seizure cases and skeletal muscle cramps (1-3). The structure-activity relationship of benzodiazepine receptor ligands has been the subject of many researches. It has been clear that at least two common features are necessary for binding benzodiazepine receptor: an aromatic ring and a coplanar proton-accepting group in a suitable distance. Moreover, the presence of a second out-of-plane aromatic ring could potentiate binding to the receptor especially for agonists (4-8). In our previous studies, we reported some new simple non-rigid derivatives of triazole and oxadiazole rings (9-18), with above-mentioned requirements for binding to the benzodiazepine receptor. Since all of our previously effective reported phenoxyphenyl oxadiazoles had halogen substitute on at list one of the phenyl rings, we synthesized a few phenoxyphenyl-1,3,4-oxadiazole derivatives, structure 1 (Figure 1), without any substitute on the phenyl rings to elucidate the role of electron withdrawing halogen in the benzodiazepine effects. Conformational analysis followed by superimposition of energy minima conformers on known benzodiazepine agonist, estazolam, was performed to clarify whether the synthesized compounds could mimic agonist structure of benzodiazepine ligands. Anticonvulsant activity of the synthesized compounds was determined as an *in-vivo *model for evaluating benzodiazepine effect.

## Experimental

*Chemistry*

Melting points were determined with a Reichert-Jung hot stage microscope and are uncorrected. ^1^H-NMR spectra were obtained with a Bruker 80 MHz spectrometer.

Chemical shifts are reported in ppm (d) values relative TMS as internal reference. Mass spectra were recorded by a Finnigan Mat TSQ-70 spectrometer. Thin-layer chromatography was carried out using Merck HF254 Silicagel. 2-Phenoxybenzoic acid was purchased from the Fluka Chemie AG. The other chemicals for synthesis were supplied from Merck (Germany).

*2-(2-Phenoxy)phenyl-1,3,4-oxadiazole-4(H)-5-tion (6)*

To a stirring solution of 4 (3.5 g , 15.4 mmol) and potassium hydroxide (1 g , 18 mmol) in ethanol 96% (85 mL) cooled in an ice bath, carbon disulfide (2.8 g, 36.8 mmole) was added drop wise. The mixture was refluxed for 7 h. The solvent was evaporated and to the residue, water was added and acidified with dilute hydrochloric acid. The precipitate was filtered and crystallized from ethanol 96% to give 3.5 g (85%) of compound 6, mp 199-201°C. IR (KBr): = 3190 cm^-1^ (NH), 1350 (C=S); 80 MHz ^1^H-NMR (CDCl_3_): δ (ppm) 6.80-7.50 (m, 8H, aromatic), 8.10 (dd, 1H, J_5,6_ = 7.1, J_4,6_ = 2.1 Hz, phenyl H_6_); MS : m/z (%) 271 (M^+^ + 1, 85), 210 (55), 197 (15), 181(100), 152 (20), 77 (5).

*2-Methylthio-5-(2-phonxy)phenyl-1,3,4-oxadiazole (7)*

To a solution of compound 6 (1 g, 3.7 mmol) in ethanol 96% (2 mL), aqueous solution of NaOH 10% (2 mL) and methyl iodide (0. 53 g , 3.73 mmol) was added and ultrasonicated for 6 min. Water was added and the solution was extracted with chloroform. The organic layer was washed with water and saturated solution of NaCl and dried with sodium sulfate and evaporated to give 1 g (95%) of compound **7 **as an oil. 80 MHz ^1^H NMR (CHCl_3_-d_1_) : δ 2.58 (s, 3H, SCH_3_), 6.80-7.60 (m, 8H, aromatic), 8.05(dd, 1H, J_5,6 _= 7.2, J_4,6_ = 2.2 Hz, phenyl H_6_). MS: m/z (%) 2.85 (M^+^ + 1, 100), 210 (5), 182 (20), 168 (10), 92 (5), 77 (10).

*5-(2-Phenoxy) phenyl-1, 3,4-oxadiazole-3(H)-2-one (8)*

To a stirring solution of compound 4 (3 g, 13.2 mmol) and triethylamine (3 mL) in dry tetrahydrofuran (150 mL), cooled in an ice bath, 1,1′-carbonyldiimidazole (4.2 g, 26.3 mmol) was added. The stirring was continued for 5 h. Triethylamine (1.5 mL) and 1,1′-carbonyldiimidazole (1.5 g, 9.4 mmol) were added and stirring was continued at room temperature overnight. The solution was evaporated and the residue was dissolved in ether and washed with hydrochloric acid 20%. A precipitate was appeared witch was filtered and crystallized from ethanol 96% to give 2.1 g (63%) of compound 8**, **mp 180-183°C. IR (KBr): ν = 3100 cm^-1^ (NH), 1790 (C=O); 80 MHz ^1^H-NMR (CDCl_3_): δ (ppm) 6.85-7.55 (m, 8H, aromatic), 8.12(dd, 1H, J_5,6_ = 7.0, J_4,6_ = 2.2 Hz, phenyl H_6_). MS: m/z (%) 254 (M^+^, 100), 197 (35), 152 (15), 120 (40).

*2-Amino-5-(2-phenoxy) phenyl-1,3,4-oxadiazole (9)*

A solution of compound 4 (6 g, 26.3 mmol) and cyanogen bromide (3.1 g, 29.3 mmol) in methanol (100 mL) was refluxed for 2 h. The solvent was evaporated and the residue was crystallized from ethanol 96% to give 5.3 g (80%) of compound 9, mp 212-214°C. IR (KBr): ν = 3280 and 3200 cm^-1^ (NH_2_), 3120 (NH dimer), 1660 (C=N); 80 MHz ^1^H-NMR (CDCl_3_): δ (ppm) 5.6 (broad s, 2H, NH2), 6.80-7.50 (m, 8H, aromatic) 8.00 (dd, 1H, J_5,6_ = 7.2, J_4,6_ = 2.1 Hz, phenyl H_6_); MS : m/z (%) 253 (M+, 100), 210 (10), 181 (35), 152 (10), 115 (5), 77 (5).

*2-(2-Phenoxy) phenyl-1,3,4-oxadiazole (10)*

To a stirring solution of compound 5 (2 g, 7.8 mmol) in dry xylene (90 mL), P_2_O_5_ (4 g) was added and refluxed for 2 h. The solution was decanted and evaporated. The residue was purified by thin layer chromatography (chloroform-methanol, 98:2) to give 1.2 g (65%) of compound 10, mp 60-62°C (ether). 80 MHz^ 1^H-NMR (CDCl3): δ (ppm) 6.80-7.60 (m, 8H, aromatic), 8.14 (dd, 1H, J_5,6_ = 8.0, J_4,6_ = 1.6 Hz, phenyl H_6_), 8.45 (s, 1H, oxadiazole); MS: m/z (%) 238 (M^+^, 100), 221 (65), 180 (55), 152 (40), 138 (5), 91 (25), 77 (35), 51 (50).

*Conformational analysis*

Conformational analysis of the designed compounds was performed by MM+ force field followed by AM1 method implemented in HyperChem 7 software (Hypercube Inc.). Global minimum forms of the compounds were superimposed on estazolam which was considered as a reference.

*Pharmacology*

Male NMRI mice (Pasteur Institute Iran) weighting 20-25 g (n = 10) were used in the experiments. The animals were kept in groups of ten in cages under constant temperature ( 24 ± 1°C ) and 12 h light/dark schedule. They had free access to standard mouse diet and tab water except during the experiment. On the day of the experiment, animals were transferred to individual cages randomly and allowed to acclimatize for 30 min before injection of drug or vehicle. Anticonvulsant activity of compounds was measured by PTZ-induced lethal convulsion test (21).

Test compounds and vehicles were administered to groups of ten mice 1 h before the injection of PTZ (100 mg/Kg, ip) and the number of dead mice were counted 30 min after injection of PTZ.

Test compounds and flumazenil (Hoffmann La Rosch) were suspended in a solution of carboxymethylcellulose (CMC) (1%) and tween 80 (0.5%) in distilled water. Diazepam ampoules (Darou Pakhsh Co.) were diluted with distilled water. Test compounds, diazepam and flumazenil were injected 1 h, 30 min, 15 min before injection of PTZ respectively. Control animals were given the corresponding vehicles. This study was conducted in accordance with protocols approved by the Institutional animal care and use committee and all experiments were performed based on the National Institutes of Health (NIH) Guide for the Care and Use of Laboratory Animals and all efforts were made to minimize the number of animals used in the study.

ED_50_ values and their 95% confidence limits were determined by computer programmed probit-regression. Fisher’s exact probability test was used to compare flumazenil treated and non-treated groups. All the data were presented as Mean (95% confidence limits) and p < 0.05 considered statistically significant.

## Results

*Chemistry*

**Figure 2:**
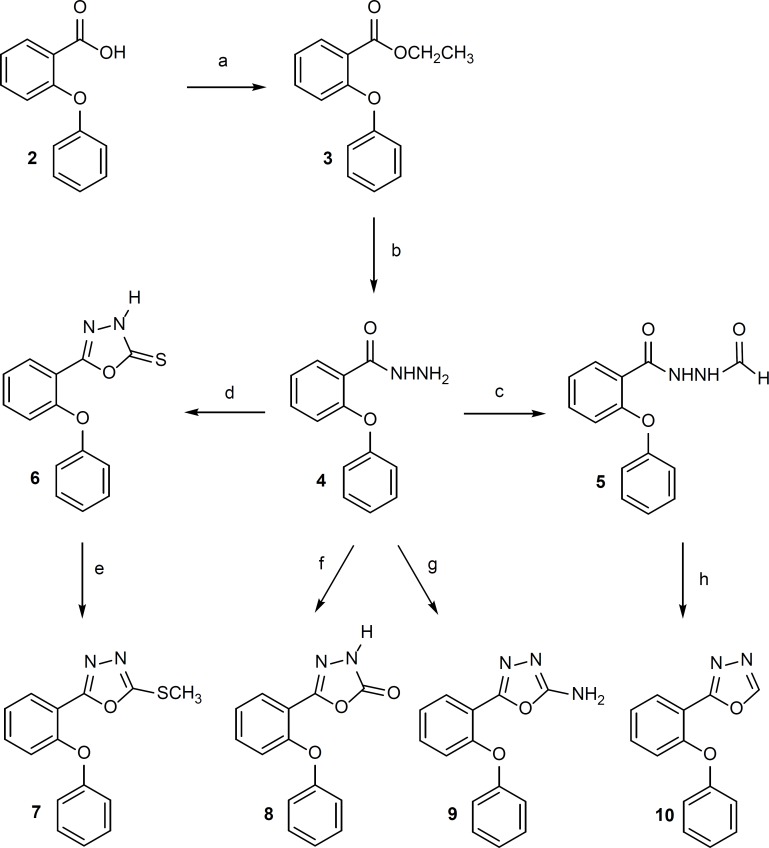
Reagents: (a) ethanol, concentrated H_2_ ; (d) CS_2_, ethanol 96%, KOH, reflux, 7 h; (e) CH_3_I, NaOH 10%, ethanol 96%, ultrasonication, room temperature, 6 min; (f) 1,1′-carbonyldiimidazole, triethylamine, dry THF, 0°C, 5 h, room temperature, 12 h; (g) BrCN, methanol, reflux, 2 h; (h) P_2_O_5_ , xylene, reflux, 2 h.

1, 3, 4-Oxadiazole derivatives 6-10 were synthesized according to Figure 2.

 Compound 3 was obtained in quantitative yield by esterification of 2-phenoxybenzoic acid with ethanol. Compound 4 was prepared in satisfactory yield from the reaction of 3 with hydrazine hydrate in ethanol at room temperature. Compound 5 was synthesized by refluxing of 4 in formic acid. 1,3,4-Oxadiazole-4(H)-5-tione ring (Compound 6) was synthesized through refluxing of 4 with carbon disulfide in alcoholic caustic potash. Methylation of 6 with methyl iodide gave **7 **in high yield. Compounds 8 and 9 were prepared in good yield by the reaction of 4 with 1,1′-carbonyldiimidazole and cyanogen bromide respectively in proper conditions. Dehydration of 5 by phosphorus pentoxide in boiling toluene gave 10 (10, 18-20). 

*Conformational analysis*

Since compounds 1 (Figure 1) are non-rigid structures, conformational optimization with MM+ force field followed by AM1 calculation was performed to confirm whether these compound could mimic proper conformation for binding to the benzodiazepine receptors. A known benzodiazepine agonist, estazolam, was considered as a reference compound and so the procedure was also performed on that. 

**Figure 3:**
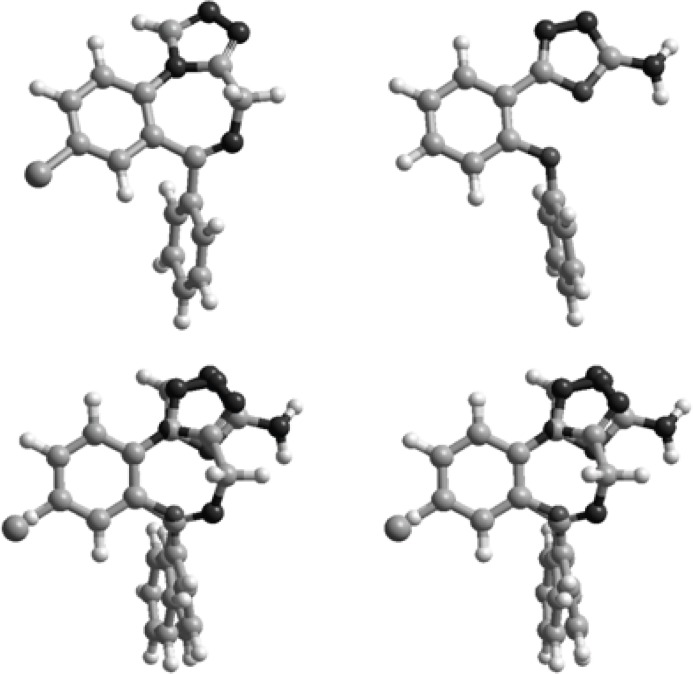
Stereoview of the superimposition of the energy minima conformers of estazolam (left) and compound 9 (right).

Superimposition of the energy minima conformers of the designed compounds on estazolam was performed to examine the matching of the benzodiazepine pharmacophores, aromatic rings and proton accepting groups (Figure 3). 

*Pharmacology*

Anticonvulsant effect of compounds was determined as a model for benzodiazepine activity through evaluation of the ability of the synthesized compounds to protect mice against a lethal dose of pentylenetetrazole (PTZ) (Table 1).

**Table 1:** Anticonvulsant activity of compounds 6-10 in PTZ-induced lethal convulsion test

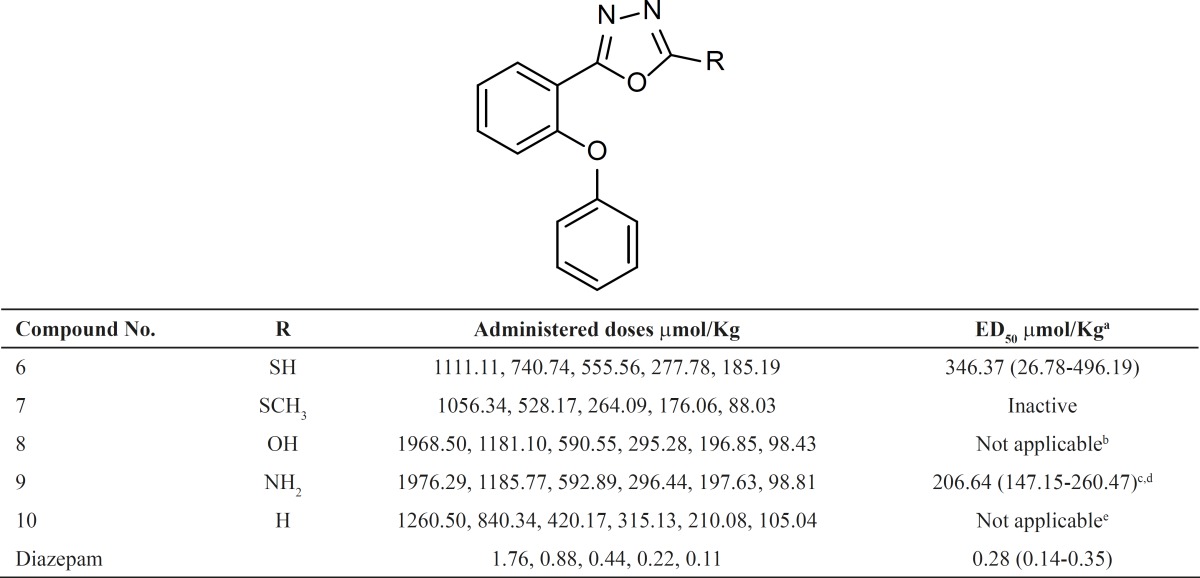

a: n = 10, 95% Confidence limits in parentheses; b: Significantly anticonvulsant but dose response relationship could not be determined; c: LD_50_ > 3000 μmol/Kg; d: ED_50_ significantly increased in the presence of flumazenil 10 mg/Kg (p < 0.05); e: In high doses was a convulsing agent

To confirm whether the anticonvulsant activity of the compounds mediates through benzodiazepine receptors, the effect of flumazenil, a benzodiazepine antagonist, in reducing the anticonvulsant activity of the compounds was determined.

## Discussion

In our previous studies, we reported phenoxyphenyl oxadiazoles with halogen substitute on at list one of the phenyl rings. In the present work, we synthesized a few phenoxyphenyl-1,3,4-oxadiazole derivatives 6-10 without any substitute on the phenyl rings to elucidate the role of electron withdrawing halogen in the benzodiazepine effects. Table 1 shows the anticonvulsant activity of compounds 6-10. Diazepam was considered as a standard benzodiazepine agonist. The results show that compound 9 with an amino substituent on 2 position of 1,3,4-oxadiazole ring has a respectable anticonvulsant activity and replacement of this group with H, OH, SH or SCH_3_ abolish or decrease the anticonvulsant effect. The fact that the activity of compound 9 is significantly reduced by flumazenil, a known benzodiazepine antagonist, confirms that this effect is mediated through benzodiazepine receptors.

Figure 2 illustrates the superimposition of energy minima conformers of compound 9 on estazolam, a known benzodiazepine agonist. It is clear that the aromatic rings and proton accepting groups, N-3 of the oxadiazole ring of compound 9 and N-2 of the triazole ring of estazolam, are well matched. These groups could be considered as the main proposed benzodiazepine pharmacophores. Therefore, compound 9 could mimic the benzodiazepine structure at the receptor sites. These results are entirely consistent with our previous findings on the other 1,3,4-oxadiazole and 1,2,4-triazole derivatives (9-18). Although compound 9 is weaker than diazepam, it should be mentioned that it has a good margin of safety and LD_50_ of this compound is bigger than 15 times of its ED_50_ (Table 1). The results are in agreement with SAR of benzodiazepine receptor ligands because in the present work we eliminated the electron withdrawing substituent in position 2 of phenoxy ring and position 4 of phenyl ring of our previously designed molecules and the anticonvulsant activity reduced. This finding confirms that the designed structures bind to the receptors as the same manner of the classic benzodiazepines. 

## References

[1] Sieghart W (1995). Structure and pharmacology of γ-aminobutyric acidA receptor subtypes. Pharmacol. Rev.

[2] Mohler H, Fritschy JM, Rudolph U (2002). A new benzodiazepine pharmacology. J. Pharmacol. Exp. Ther.

[3] Sieghart W, Ernst M (2005). Heterogeneity of GABAA receptors: revived interest in the development of subtype-selective drugs. Curr. Med. Chem. Central Nervous Syst. Agents.

[4] Clayton T, Chen JL, Ernst M, Richter L, Cromer BA, Morton CJ, Ng H, Kaczorowski CC, Helmstetter FJ, Furtmüller R, Ecker G, Parker MW, Sieghart W, Cook JM (2007). An updated unified pharmacophore model of the benzodiazepine binding site on γ-aminobutyric acida receptors: correlation with comparative models. Current Medicinal Chemistry.

[5] Zhang W, Koehler KF, Zhang P, Cook JM (1995). Development of a comprehensive pharmacophore model for the benzodiazepine receptor. Drug Des. Discov.

[6] Filizola M, Harris DL, Loew GH (2000). Development of a 3d pharmacophore for nonspecific ligand recognition of alpha1, alpha2, alpha3, alpha5, and alpha6 containing GABAA/benzodiazepine receptors. Bioorg. Med. Chem.

[7] Huang Q, He X, Ma C, Liu R, Yu S, Dayer CA, Wenger GR, McKernan R, Cook JM (2000). Pharmacophore/receptor models for gaba(a)/bzr subtypes (alpha1beta3gamma2, alpha5beta3gamma2, and alpha6beta3gamma2) via a comprehensive ligand-mapping approach. J. Med. Chem.

[8] Lager E, Nilsson J, Ostergaard Nielsen E, Nielsen M, Liljefors T, Sterner O (2008). Affinity of 3-acyl substituted 4-quinolones at the benzodiazepine site of gaba(a) receptors. Bioorg. Med. Chem.

[9] Akbarzadeh T, Tabatabai SA, Khoshnoud MJ, Shafaghi B, Shafiee A (2003). Design and synthesis of 4h-3-(2-phenoxy)phenyl-1,2,4-triazole derivatives as benzodiazepine receptor agonists. Bioorg. Med. Chem.

[10] Almasirad A, Tabatabai SA, Faizi M, Kebriaeezadeh A, Mehrabi N, Dalvandi A, Shafiee A (2004). Synthesis and anticonvulsant activity of new 2-substituted-5- [2-(2-fluorophenoxy)phenyl]-1,3,4-oxadiazoles and 1,2,4-triazoles. Bioorg. Med. Chem. Lett.

[11] Zarghi A, Faizi M, Shafaghi B, Ahadian A, Khojastehpoor HR, Zanganeh V, Tabatabai SA, Shafiee A (2005). Design and synthesis of new 2-substituted- 5-(2-benzylthiophenyl)-1,3,4-oxadiazoles as benzodiazepine receptor agonists. Bioorg. Med. Chem. Lett.

[12] Zarghi A, Tabatabai SA, Faizi M, Ahadian A, Navabi P, Zanganeh V, Shafiee A (2005). Synthesis and anticonvulsant activity of new 2-substituted-5-(2- benzyloxyphenyl)-1,3,4-oxadiazoles. Bioorg. Med. Chem. Lett.

[13] Almasirad A, Vousooghi N, Tabatabai SA, Kebriaeezadeh A, Shafiee A (2007). Synthesis, anticonvulsant and muscle relaxant activities of substituted 1,3,4-oxadiazole, 1,3,4-thiadiazole and 1,2,4-triazole. Acta Chimica Slovenica.

[14] Foroumadi A, Sheibani V, Sakhteman A, Rameshk M, Abbasi M, Farazifard R, Tabatabai SA, Shafiee A (2007). Synthesis and anticonvulsant activity of novel 2-amino-5-[4-chloro-2-(2- chlorophenoxy) phenyl]- 1,3,4-thiadiazole derivatives. Daru.

[15] Zarghi A, Hajimahdi Z, Mohebbi S, Rashidi H, Mozaffari S, Sarraf S, Faizi M, Tabatabaee SA, Shafiee A (2008). Design and synthesis of new 2-substituted- 5-[2-(2-halobenzyloxy)phenyl]-1, 3,4-oxadiazoles as anticonvulsant agents. Chem. Pharm. Bull.

[16] Zarghi A, Hamedi S, Tootooni F, Amini B, Sharifi B, Faizi M, Tabatabai SA, Shafiee A (2008). Synthesis and pharmacological evaluation of new 2-substituted-5-{2-[(2- halobenzyl)thiophenyl}-1,3,4-oxadiazoles as anticonvulsant agents. Sci. Pharm.

[17] Mahdavi M, Akbarzadeh T, Sheibani V, Abbasi M, Firoozpour L, Tabatabai SA, Shafiee A, Foroumadi A (2010). Synthesis of two novel 3-amino-5-[4-chloro-2- phenoxyphenyl]-4h-1,2,4-triazoles with anticonvulsant activity. Iranian J. Pharm. Res.

[18] Faizi M, Sheikhha M, Ahangar N, Tabatabaei Ghomi H, Shafaghi B, Shafiee A, Tabatabai SA (2012). Design, Synthesis and Pharmacological Evaluation of Novel 2-[2-(2- Chlorophenoxy)phenyl]-1,3,4-oxadiazole Derivatives as Benzodiazepine Receptor Agonists. Iranian J. Pharm. Res.

[19] Firoozi F, Javidnia K, Kamali M, Fooladi A, Foroumadi A, Shafiee A (1995). Syntheses of substituted 1-methyl-2-(1,3,4-thiadiazol-2-yl)-4-nitropyrroles and 1-methyl-2-(1,3,4-oxadiazol-2-yl)-4-nitropyrroles. J. Heterocycl. Chem.

[20] Shafiee A, Naimi E, Mansobi P, Foroumadi A, Shekari M (1995). Syntheses of substituted-oxazolo-1,3,4- thiadiazoles, 1,3,4-oxadiazoles, and 1,2,4-triazoles. J. Heterocycl. Chem.

[21] Morpugo C (1971). A new design of the screening of CNS-active drugs in mice (a multidimensional observation procedure and the study of pharmacological interactions). Arzneim. Forsch. (Drug Res.).

